# Development of novel low-cost isolator UMELI (unbonded mesh elastomeric layered isolator): experimental investigations

**DOI:** 10.1038/s41598-024-70927-0

**Published:** 2024-08-27

**Authors:** Zoheb Nawaz Md., Mohan S C

**Affiliations:** Department of Civil Engineering, BITS Pilani Hyderabad Campus, Hyderabad, Telangana 500078 India

**Keywords:** Seismic isolation, Experimental investigation, Unbonded mesh elastomeric layered isolator, Unreinforced elastomeric isolator, Engineering, Civil engineering

## Abstract

Seismic isolation is a highly efficacious method for reducing the seismic load on structures. This technique is widely adopted to safeguard structures from earthquakes. Despite the promising results demonstrated by numerous isolation techniques, their implementation remains a significant challenge, particularly in developing countries, due to the high costs associated with manufacturing. Therefore, a novel and affordable base isolation technique has been proposed, namely unbonded mesh elastomeric layered isolator (UMELI). UMELI consists of steel mesh sandwiched between the unbonded layers of elastomers, resulting in an affordable isolator to be used for lightweight, low-rise structures. One of the crucial characteristics of this novel isolator is that it does not require a specialised manufacturing process unlike other elastomeric isolators. In the present study, experimental investigations are conducted to evaluate the dynamic characteristics such as dynamic vertical stiffness, equivalent lateral stiffness, and equivalent viscous damping ratio of UMELI. Its characteristics were studied for different layered isolators and are compared with the unreinforced elastomeric isolator. The investigations have demonstrated the effectiveness of the UMELI by increasing its vertical stiffness and reducing lateral stiffness, thereby enhancing the isolation period with the addition of a steel mesh layer.

## Introduction

Unreinforced masonry (URM) structures are commonly constructed buildings that are typically located in rural regions of developing countries. This is attributed to their cost-effectiveness and their construction simplicity^1^. When this URM was designed, it was specifically focused on withstanding gravity loads. The structure does not possess the necessary structural system to endure horizontal forces. Therefore, these URM structures are extremely susceptible to earthquakes. It is estimated that approximately 75% of earthquake-induced fatalities in the past century have resulted from the collapse of masonry buildings^2^. Situated within numerous seismically active regions of developing countries, URM buildings often lead to significant social and economic losses owing to structural damage or collapse during earthquakes^3,4^. The utilization of locally available materials, traditional construction techniques, and the absence of standardized construction practices are considered to be the primary factors contributing to the inadequate seismic performance of URM structures^5,6^. Several developing countries do not follow the recommendations outlined in earthquake codes during the URM construction^7,8^. Such constructions are a major threat to people during earthquakes, thus highlighting the need for an effective technique to mitigate the vulnerability of URM buildings.

Traditional seismic-resistant design techniques basically depend on the structural strength to withstand seismic forces, stiffness to control the displacement of the structure, and ductility to allow for sufficient deformation without failure^9^. This traditional construction requires enhancing the strength and stiffness of the masonry, which in turn increases the overall cost of the structure. Along with that, it also attracts more inertia, which can be perilous for both the occupants and the contents. An alternative to this traditional technique is to utilize base isolation techniques, which can reduce the inertial load on a structure. These techniques decouple the vibration of the structure from the ground, resulting in a decrease in lateral force exerted on the structure. As a result, the force and deformation requirements on the structural components are reduced^10–17^.

In light of the benefits of utilizing base isolation technology, a number of researchers have recently examined the mechanical properties of cost-effective isolation devices and endeavored to create constitutive models to describe their lateral response^18–21^. Among them are Recycled rubber elastomeric isolator^24^, annular unbonded elastomeric isolators^22,23^, and low-cost scrap tire pads^25^. Affordable isolation bearings with decreased displacement capacities are also appealing in densely populated urban areas where it is not feasible or very costly to create seismic gaps as required by building codes^26^. Authors have recently investigated the behavior of the Unreinforced Elastomeric Isolator (UEI)^27–29^. The isolator has shown effective behavior in terms of lateral stiffness and equivalent viscous damping ratio. However, when compared with the other isolators, the vertical stiffness was several times lesser. This low vertical stiffness has produced rocking motion during the time history analysis of the masonry model in ABAQUS. Even though the stresses developed due to the rocking motion are within the capacity of the masonry, it still does not satisfy the serviceability criteria. To overcome this drawback, the authors of this paper have developed a novel low-cost base isolation technique, namely an unbonded mesh elastomeric layered isolator (UMELI). UMELI consists of steel mesh sandwiched between the unbonded rubber layers, resulting in a lightweight, cost-effective isolator for use in low-rise residential buildings. While previous research has demonstrated the effectiveness of steel mesh-reinforced elastomeric isolation bearings for bridges, these bearings are bonded between the steel mesh and elastomeric layers^30,31^. A key feature of the UMELI is that it does not require specialized manufacturing, as the rubber layers and steel mesh of the desired thickness can be readily sourced from stores. This makes UMELI particularly suitable for widespread use in underdeveloped rural regions.

Initially, the overall design of the UMELI is demonstrated along with its manufacturing process. Subsequently, the performance of the innovative isolator with varying numbers of layers is experimentally evaluated to characterize mechanical behavior such as dynamic vertical stiffness, lateral stiffness, and equivalent viscous damping ratio. The results are then compared with the unreinforced elastomeric isolator.

## Design of unbonded mesh elastomeric layered isolator

The proposed isolation technique is envisioned to be used for lightweight structures such as URM buildings. Therefore, the UMELI was specifically designed for a single-story URM building, a common structure often seen in developing nations such as India. The masonry building's overall weight was determined to be 300 kN^32^. The structure is considered to be resting on six isolators, where each isolator is engineered to endure a compressive load of 50 kN. To evaluate the equivalent lateral stiffness (${K}_{h}$), it requires mass ($M$) acting on each isolator and the target time period ${(T}_{h})$. In this case, ${T}_{h}$ is assumed to be 1.6 s at a shear strain of 25%, and the $M$ is the weight acting on the isolator, i.e., 5 tonnes. Then, using Eq. (1), the lateral effective lateral stiffness is calculated.

1 $${K}_{h}=M\cdot {\left(\frac{2\pi }{{T}_{h}}\right)}^{2}$$

The lateral stiffness of the isolator is calculated to be 77.11 N/mm using Eq. (1). To determine the geometric parameters of the isolator, various researchers have developed analytical models that incorporate the warping deformation of fiber and the detachment of the contact area of the isolator from the surface during roll over. Initially, an equation for the effective shear modulus $( {G}_{eff}$) was proposed (Eq. 6), which considers the effect of vertical pressure on the shear modulus^33,34^. In another study, the reduction in contact area during rollover deformation was considered while maintaining the shear modulus constant. This led to the proposal of an equation for the effective contact area (${A}_{eff}$), which includes the projected length (*d*) (Eqs. 4 and 5)^35,36^. Similarly, another equation was proposed to calculate the effective contact area, where the reduced area is calculated as the product of the isolator's side length and the perpendicular side length minus the projected length (Eq. 3)^36^. Finally, an expression was developed that incorporates both the effective shear modulus and effective area (Eq. 2)^36^.

Assuming the plan area ($$A)$$ of the isolator as 70 mm × 70 mm, the shear modulus as 0.98 MPa ($$G$$) and initial thickness of the rubber ($${t}_{r})$$ as 15 mm, then the total height of the isolator ($$h$$) is calculated using following equations.

2 $${K}_{h}=\frac{{G}_{eff}\cdot {A}_{eff}}{{t}_{r}}$$

3 $${A}_{eff}=a(a-d)$$

here *a* is the length of the isolator

4 $$d=\frac{25}{16} \gamma h$$

$\gamma$ refers to the constant that relates the projected length and it is given by Eq. (5). In this study, the $\gamma$ is assumed to be 0.157 to simplify the expression and obtain the total height of the isolator.

5 $$u=\frac{25}{64}h\left[2\gamma \sqrt{1+4{\gamma }^{2}}+\text{ln}\left(2\gamma +\sqrt{1+4{\gamma }^{2}}\right)\right].$$

6 $${G}_{\text{eff }}=G\left[1-{\left(\frac{{p}_{z}}{{p}_{\text{crit },0}\left(1-{\left(\frac{u}{a}\right)}^{2}\right)}\right)}^{2}\right]\left(1-\frac{u}{a}\right).$$

In the Eq. (6), $${P}_{crit,0}$$ represents the critical stress of the isolator and is defined by $${P}_{crit,0} =\frac{{P}_{crit}}{{a}^{2}}$$. The critical load, $${P}_{crit}$$, is given by $${P}_{crit}=\frac{\sqrt{2}\pi GASr}{{t}_{r}}$$, where $$r=\frac{a}{2\sqrt{3}}$$ is the radius of gyration, $$G$$ is the shear modulus obtained from the manufacturer, and *u* is the given lateral displacement. Given these values, the critical load of the isolator ($${P}_{crit})$$ is determined to be 55 kN, and the $${G}_{\text{eff}}$$ is found to be 0.26 MPa. Using the obtained lateral stiffness and effective shear modulus and by rearranging Eqs. (2)–(4), the total height of the isolator is calculated to be 31.17 mm. For each of the manufacturing, this was rounded to 30 mm. In the current study, the performance of the UMELI is evaluated with varying number of layers of steel mesh. Hence, with the dimensions obtained, the primary shape factor *S*_*1*_ $$({S}_{1}=\frac{a}{4{t}_{r}})$$ of UEI is determined to be 0.58 and for 2-layered and 3-layered UMELI it is determined to be 1.16 and 1.75. The secondary shape *S*_*2*_ $$({S}_{2}=\frac{a}{h})$$ remains same for all the cases i.e., 2.33^37–40^.

## Manufacturing process of unreinforced mesh elastomeric layered isolator

**Fig. 1:**
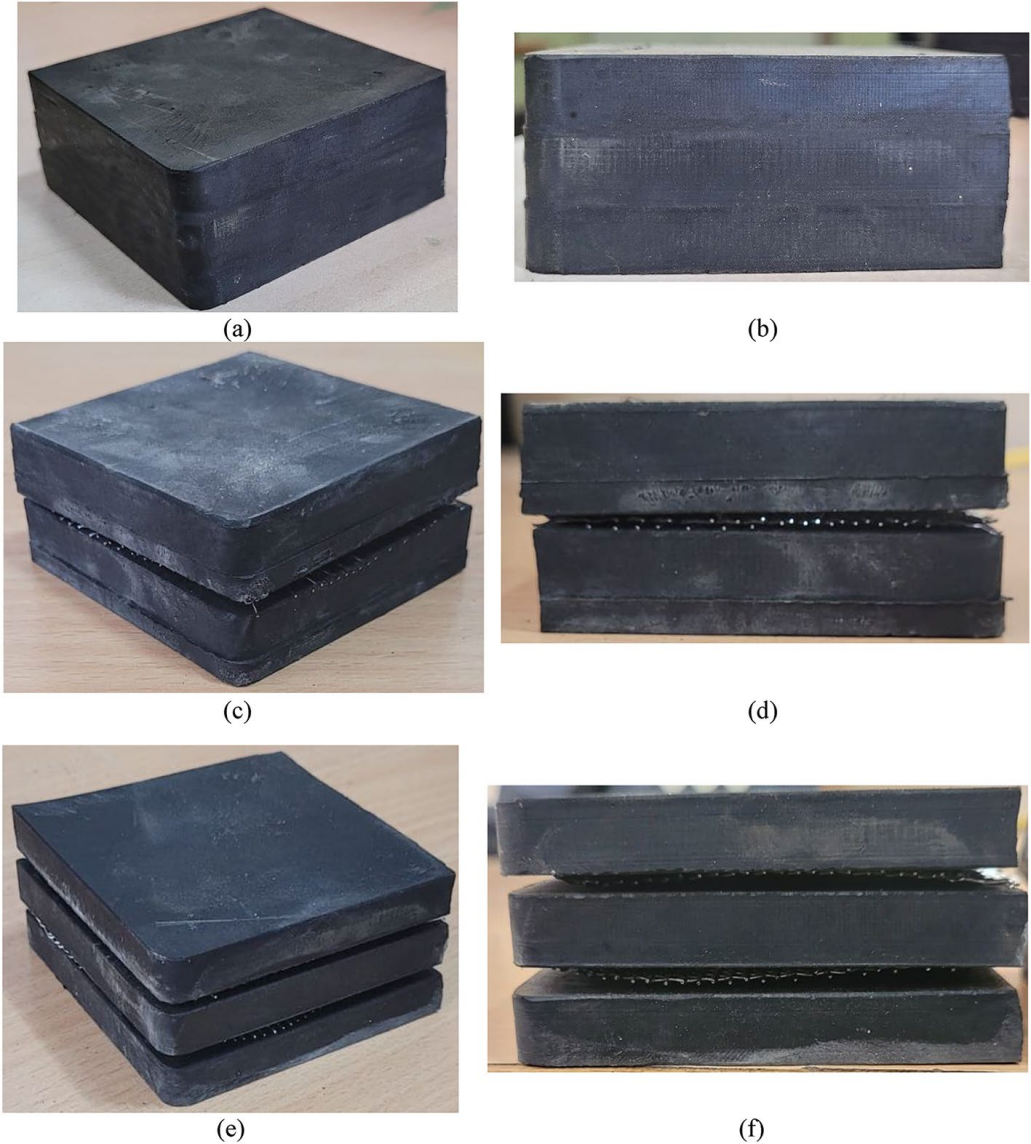
Different view of the isolators (**a**, **b**): UEI, (**c**, **d**): 2-layered UMELI, (**e**, **f**): 3-layered UMELI.

Materials with simple and adaptable manufacturing technologies are best suited to the task of producing low-cost seismic isolators. As a result, the researchers contacted a nearby supplier named Kumar Rubbers to manufacture a rubber sample measuring 70 × 70 mm, with thicknesses of 10 mm and 15 mm and with a shore A hardness of 40 (equivalent to a shear modulus of 0.98 MPa). The rubber isolator adhered to the established manufacturing procedures. RMA 5 (Rubber Manufacturing Association compound), sulfur, zinc oxide, and stearic acid are among the compounds employed in the mastication process. After 40 min of masticating process, the mixture is subjected to vulcanization in a rectangular mold measuring 500 × 500 mm of plan dimensions at a temperature of 110 degrees Celsius for 10 min. A cutting machine is used to precisely cut the material to the dimensions of 70 × 70 mm (refer to Fig. 1). The same process is employed for different thicknesses. This isolator utilized a 0.5 mm thick woven steel mesh of SS 304 grade. The tensile strength of the mesh is obtained to be 250MPa. The mesh was cut into 70 × 70 mm dimensions and arranged in an alternating pattern with the rubber layer, as depicted in Fig. 1. The physical properties of the isolators with varying thicknesses have been tabulated in Table 1. This technique is unique because the steel mesh and rubber are not bonded or vulcanised together. The cost associated with each UEI is $3.50 USD. In contrast, the costs for 2-layered and 3-layered UMELI are $3.75 USD and $4.00 USD, respectively. These costs include the expense of the steel mesh incorporated in the UMELI. Unlike the strip isolation technique^38,41^, which does not require a plinth beam for load transfer to the isolator, this method necessitates the inclusion of an additional concrete beam to facilitate effective load transfer.

**Table 1:** Physical properties of the isolators.

Isolator	Overall Thickness (mm)	Thickness of the rubber (mm)	No. rubber layers	No. of steel mesh layers
UEI	30.23	30.23	1	0
UMELI—2 layers	33.51	15	2	1
UMELI—3 layers	34.20	10	3	2

## Experimental investigation

Experimental tests were conducted on the UEI and UMELI to assess their performance under compression and shear. A cyclic compression test was conducted on the isolators to assess their dynamic vertical stiffness. In contrast, a cyclic shear test was performed to determine the effective lateral stiffness and equivalent viscous damping ratio of the isolators.

### Cyclic compression test

**Fig. 2:**
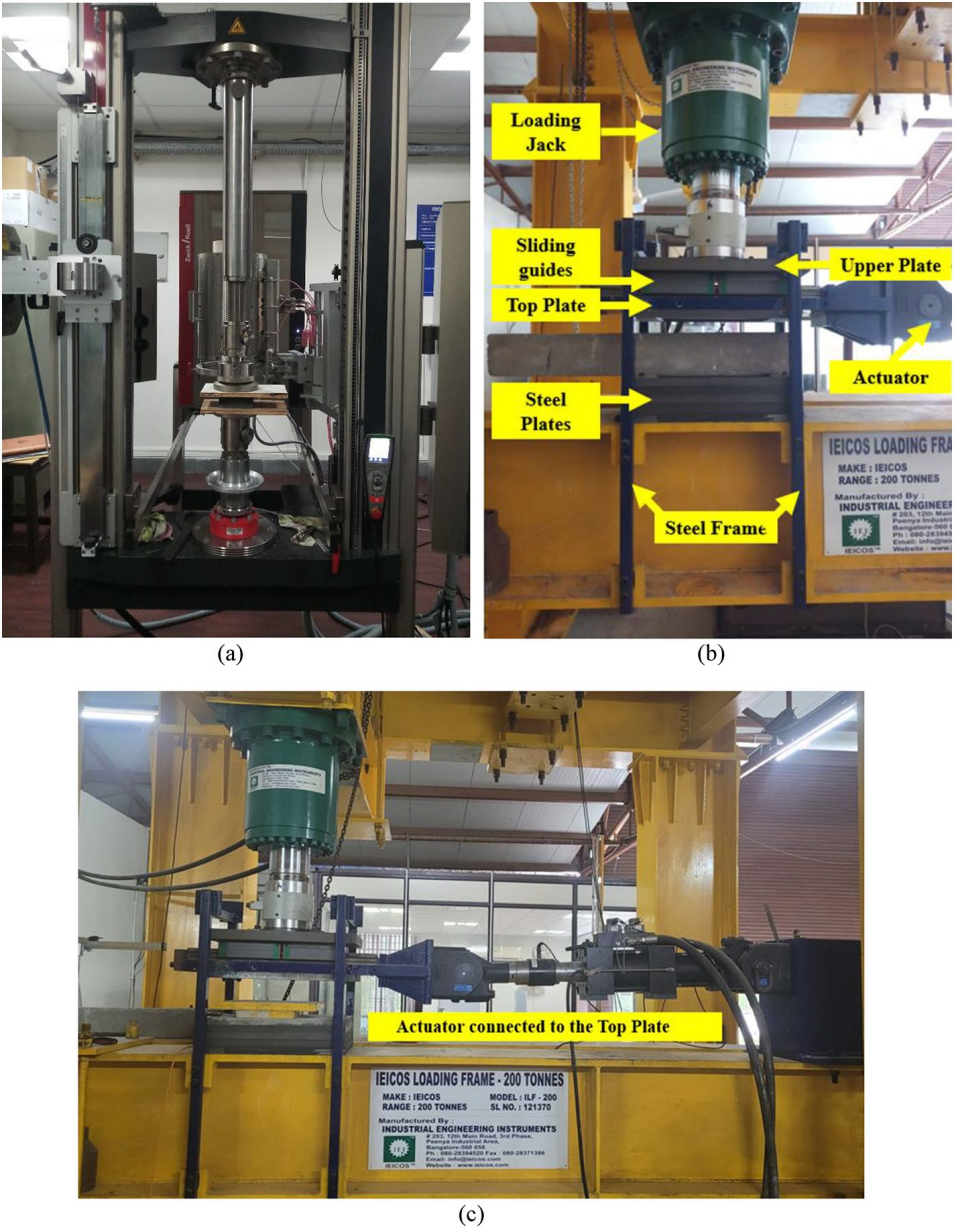
(**a**) Zwick Z100 UTM, (**b**, **c**) arrangement for Shear cyclic test.

**Fig. 3:**
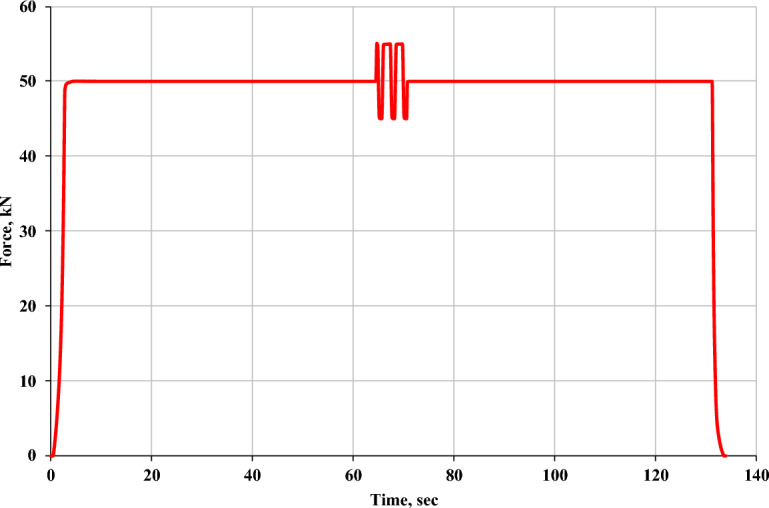
Loading profile for the compressive test.

The UMELI and UEI isolator samples have been tested for their dynamic vertical stiffness with a Zwick 100 universal testing machine (see Fig. 2a). The machine's stroke length is 1450 mm, and it can exert a maximum force of 100 kN. The samples were compressed under a static load until reaching the specified design load. A 60-s pause was implemented with the design load to accommodate the viscoelastic effect. Subsequently, three cycles with an amplitude equivalent to 10% of the design load were imposed. After 60 s, the samples were unloaded at a rate of 500 mm/min. The profile of the force applied during the test is shown in Fig. 3.

Vertical stiffness is one of the essential properties of an isolator, which determines the relationship between deformation and applied load along the vertical axis. After an isolation system is installed, it's crucial that the vertical stiffness be consistent and reliable. Differential settlements caused by variations in vertical stiffness could compromise the structure even without seismic activity. The isolators were subjected to cyclic loads to evaluate the vertical stiffness (${K}_{v}$), and Eq. (7) is used to obtain the values.

When the forces ${F}_{v}^{+}$ and ${F}_{v}^{-}$ are applied to the isolator, the corresponding displacements are ${\Delta }_{v}^{+}$ and ${\Delta }_{v}^{-}$ were recorded and these values are used to calculate vertical stiffness (${K}_{v}$).

7 $${K}_{v}=\frac{\left|{F}_{v}^{+}\right|+\left|{F}_{v}^{-}\right|}{\left|{\Delta }_{v}^{+}\right|+\left|{\Delta }_{v}^{-}\right|}$$

Following the calculation of the vertical stiffness, the vertical frequency (${f}_{v}$) were calculated using Eq. (8). To obtain the vertical frequency, it requires the vertical pressure ($P$) applied on the isolation, along with the area and acceleration due to gravity $(g)$.

8 $${f}_{v}=\frac{1}{2\pi }\sqrt{\frac{{K}_{v}\cdot g}{P\cdot A}}$$

The isolators' dynamic vertical stiffness was determined by calculating the tangent slope of the loading's cyclic portions. Table 2 compares the variation of the dynamic vertical stiffness for the UEI and UMELI with varying number of layers. As rubber is highly nonlinear when the load is applied on the isolator, the voids present in the rubber close and offer resistance. This phenomenon is usually known as the run-in effect. Due to this run-in effect, the vertical stiffness of UEI is high. It is clearly observed that the dynamic vertical stiffness of the UMELI is greater than that of the UEI (see Table 2).

**Table 2:** Comparison of dynamic vertical stiffness for UEI and UMELI.

Isolator	Thickness (mm)	Static deformation (mm)	Vertical stiffness, ${{\varvec{K}}}_{{\varvec{v}}}$ (kN/mm)	Vertical Time Period, 1/${{\varvec{f}}}_{{\varvec{v}}}$ (s)	Vertical Natural frequency, ${{\varvec{f}}}_{{\varvec{v}}}$ (Hz)
UEI	30.23	20.88	19.86	0.10	10.03
UMELI—2 layers	33.51	21.88	23.64	0.09	10.94
UMELI—3 layers	34.20	22.10	37.68	0.07	13.82

**Fig. 4:**
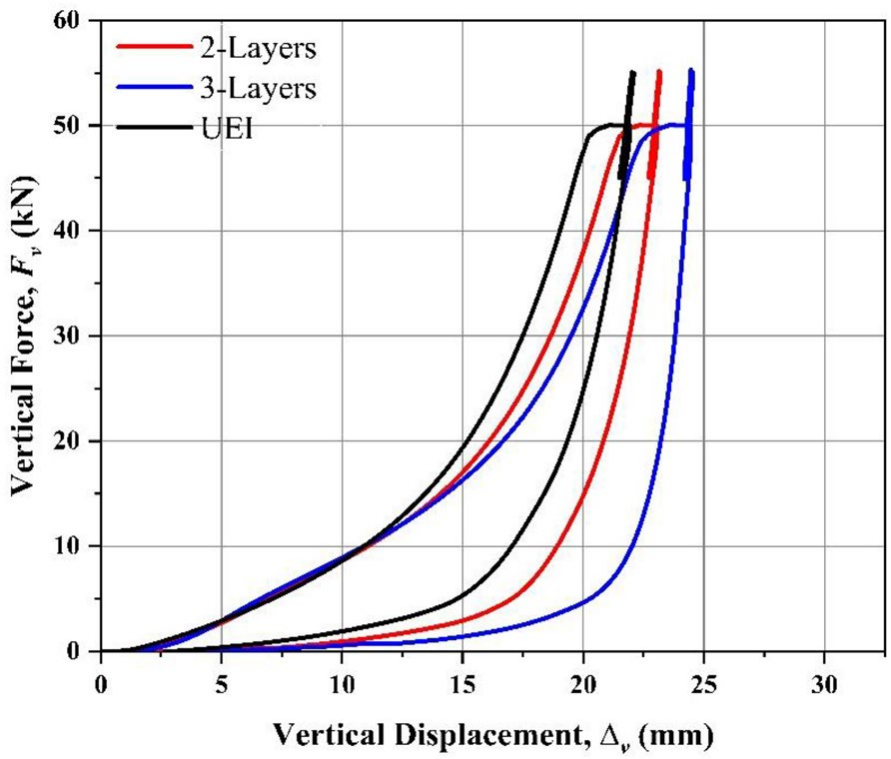
Force–displacement curves obtained from the compressive test.

**Fig. 5:**
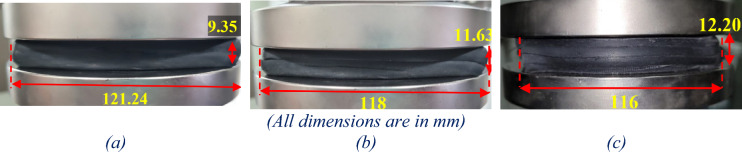
Comparison of vertical deformation (**a**) UEI; (**b**) 2-layered UMELI; (**c**) 3-layered UMELI.

Even in the UMELI, the vertical stiffness increases with the increase in the number of steel mesh layers. This is due to the confinement of the bulging in the rubber by the steel mesh. As there is no bonding between the elastomer and the steel mesh, the confinement of the elastomer's bulging is solely based on the frictional interaction between the elastomer and the steel mesh. Upon the application of vertical pressure to the UMELI, the gaps in the steel mesh are filled with the elastomer. The steel wires resist the bulging slot by slot, which, in turn, provides overall confinement to the bulging. Once the tensile stress generated by the elastomer on the steel mesh exceeds the frictional force between the steel wires, internal sliding occurs, resulting in partial confinement. The more the number of the steel mesh layers, the more the resistance offered to the bulging of rubber, which tends to increase the vertical stiffness. The force–displacement curves obtained from tests have been shown in Fig. 4. Figure 5 depicts the vertical deformation of the isolator during the compression test for UEI and UMELI. Even though the dynamic stiffness is high enough, higher flexibility is observed in the vertical direction under static loads, i.e., a static deformation of 20.88 mm was measured for the UEI. Meanwhile, for the UMELI—2 layers, it was 21.88 mm; for the UMELI—3 layers, it was 23.10 mm. It should be noted that the initial thickness of the isolators is not the same in all the cases. With the increase in the number of mesh layers, the overall thickness of the isolator increases accordingly. In order to make these isolators comparable, the UMELI’s thickness has been normalized to UEI, i.e., the excess thickness measured in the UMELI has been removed from both the thickness and the static deformation. Hence, the corrected static stiffness of the UMELI is 18.60 mm and 18.13 mm for 2 layers and 3 layers.

From Table 2, the vertical stiffness of the UMELI—3 layers has increased by 47% compared to UEI. Similarly, an increase in vertical stiffness of 15% was observed for UMELI—2 layers compared to UEI. The increase in the vertical stiffness of UMELI compared to UEI shows the effectiveness of the proposed technique in terms of vertical stiffness. The following sections discuss the experimental characterization of isolators in the lateral direction. As the shape factor increases from 0.58 to 1.16 (2-layered UMELI), there is a 19% increase in vertical stiffness. Furthermore, when the shape factor increases from 0.58 to 1.75 (3-Layered UMELI), a 90% increase in vertical stiffness is observed.

### Shear test with varying amplitude

**Fig. 6:**
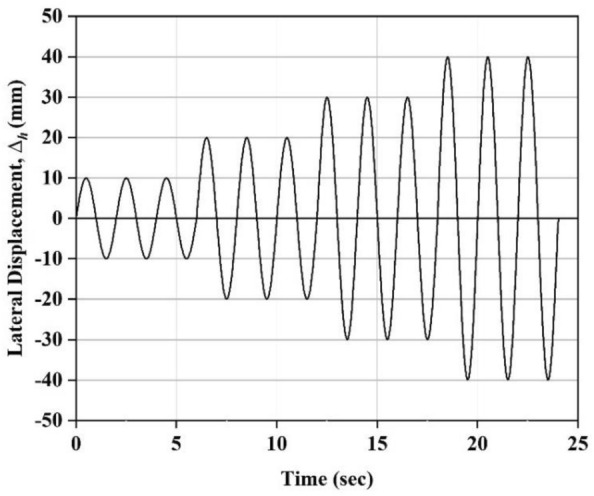
Amplitude profile with varying shear strain.

Shear cyclic experiment on isolators is a reliable technique for precisely measuring the stiffness and damping ratio. In this test, Isolators are subjected to a series of controlled horizontal displacement and vertical load cycles. The test was carried out with equipment shown in Fig. 2b,c. Additionally, it is important to note that the tests were carried out at room temperature, and 200 samples were recorded every second. In order to test the shear cyclic behavior, the isolator was put under constant vertical pressure while it was moved horizontally in a cyclic pattern. The cyclic displacement was given in a sinusoidal waveform with three full cycles. The displacement amplitude has been varied in increased order, such as ± 25%, ± 50%, ± 75%, and ± 100% of the thickness of the isolator as shown in Fig. 6.

Equation (9) is used to calculate the effective lateral stiffness (*K*_*h*_) of the isolator for each cycle of the test.

9 $${K}_{h}=\frac{\left|{F}_{h}^{+}\right|+\left|{F}_{h}^{-}\right|}{\left|{\Delta }_{h}^{+}\right|+\left|{\Delta }_{h}^{-}\right|}$$

here, ${\Delta }_{h}^{+}$ and ${\Delta }_{h}^{-}$ represents the applied displacement to the isolator and ${F}_{h}^{+}$ and ${F}_{h}^{-}$ are forces recorded during the test. Equation (10) is used to calculate the equivalent viscous damping (*ξ*) of the isolator.

10 $$\xi = \frac{{W}_{d}}{2\pi {K}_{h}{{\Delta }_{max}}^{2}}$$

where ${W}_{d}$ is the area under the curve obtained from the test and ${\Delta }_{max}$ denotes the maximum displacement applied during the test.

As the isolation period of the isolator is the crucial parameter in the design, following the calculation of the equivalent lateral stiffness $({K}_{h})$, the isolation period of the isolator (${T}_{h}$) is calculated using Equation (11).

11 $${T}_{h}=2\pi \sqrt{\frac{P\cdot A }{{K}_{h}\cdot g}}$$

Equation (11) can also be written in terms of shear modulus (*G*). The modified equation is shown in Eq. (12).

12 $${T}_{h}=2\pi \sqrt{\frac{P\cdot {t}_{r}}{G\cdot g}}$$

**Fig. 7:**
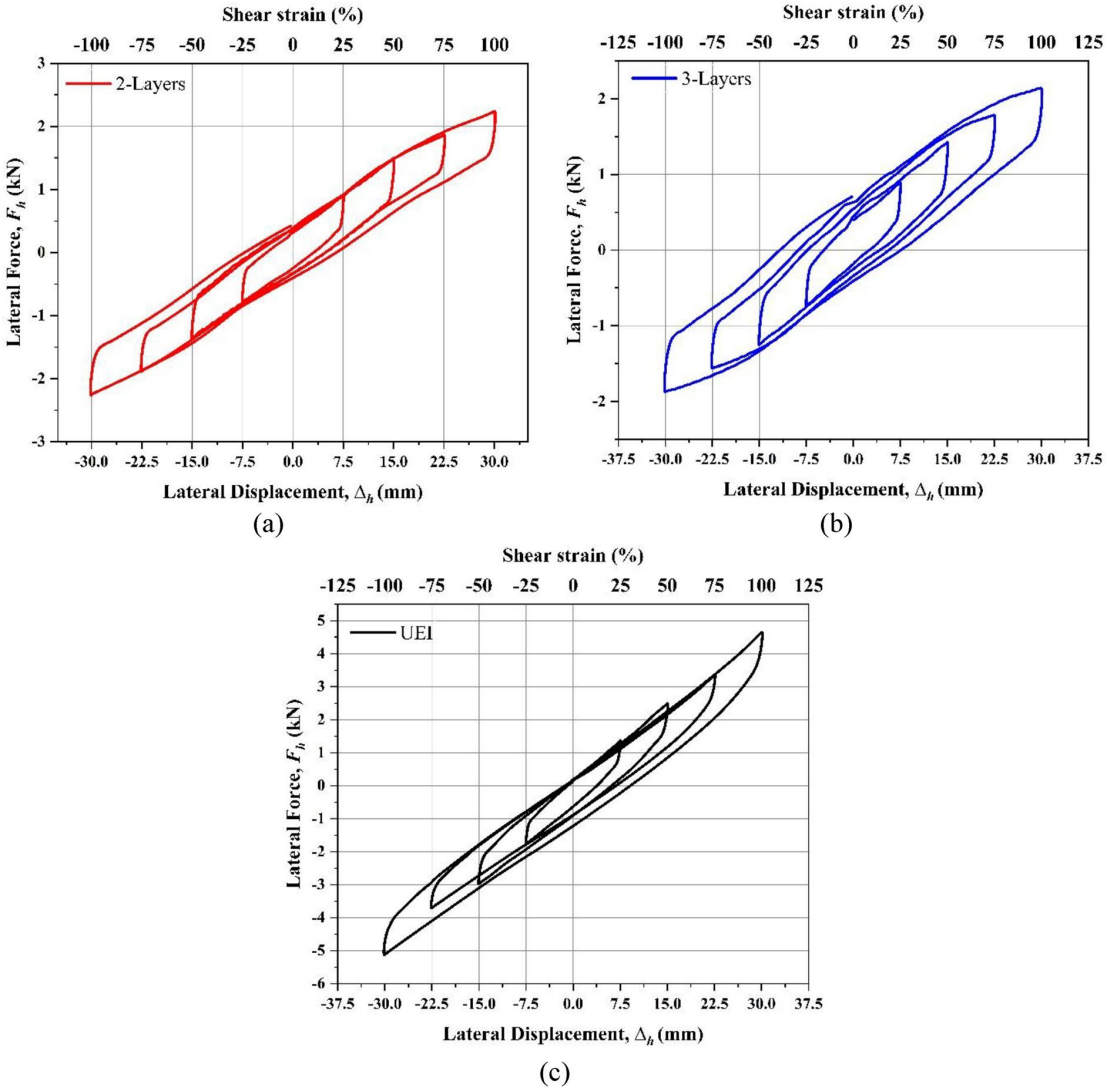
Force–displacement curves obtained from the shear cyclic test (**a**) 2-layered UMELI. (**b**) 3-layered UMELI and (c) UEI.

**Fig. 8:**
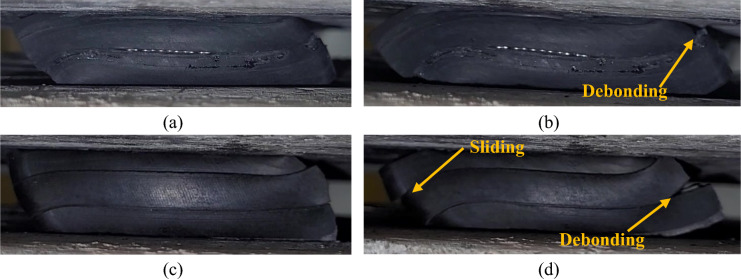
Close up view of the isolator at 50% (**a**, **c**) and 100% (**b**, **d**) strain. (**a**, **b**): 2-layered UMELI, (**c**, **d**): 3-layered UMELI.

An ascending loading path was employed to examine the impact of varying strain levels on the lateral response of the isolator, as illustrated in Fig. 6. Figure 7 depicts the Lateral force–displacement curves obtained from the test for different cases. The two crucial mechanical properties of the isolator, i.e., Equivalent lateral stiffness (*K*_*h*_) and the equivalent viscous damping ratio (ξ), are evaluated from the force–displacement curves obtained. As the three cycles are obtained from the test, *K*_*h*_ and ξ are calculated for the third cycle to reduce the relaxation effect. *K*_*h*_ is determined by considering the third cycle's maximum and minimum lateral forces for the given displacement. Similarly, calculating the area under the third curve determines the viscous damping ratio. Similar procedure has been repeated for various strain levels. These two crucial parameters were obtained using Eqs. (9) and (10). Figure 8 shows the deformed views of isolators at various shear strain levels. For all the cases, during the experiment, the isolators were observed to have returned to their original position.

### UEI

A shear cyclic test was performed on the UEI with progressively increasing shear strain under a vertical compressive force of 50 kN. The force–displacement curve from this test is illustrated in Fig. 7a. As indicated in Fig. 10a,b, there is a noticeable decrease in both *K*_*h*_ and *ξ* as shear strains increase. For example, when shear strains rise from 25 to 100%, the *ξ* drops from 14.24 to 6.37%, and the *K*_*h*_ reduces from 0.206 to 0.155 kN/mm. This decline in *K*_*h*_ with higher shear strain is due to the material's softening behavior, which is associated with the ends of the isolator rolling over. During this, the ends of the isolator are detached from the contact surfaces. At 100% shear strain, the horizontal stiffness (*K*_*h*_) is notably 128 times lower than the previously measured vertical stiffness (*K*_*v*_) from compression tests. The decreasing trend in the damping ratio shown similar behavior with High Damping Rubber fibre-reinforced elastomeric isolator^21,28^. This can be attributed to the rubber compound used in the study.

### 2-layer UMELI

2-layer UMELI showed a similar trend to that of the UEI. With the increase in the shear strain, the *K*_*h*_ and *ξ* decrease, as shown in Fig. 10a,b. The *ξ* reduced from 19.26 to 9.13%, whereas *K*_*h*_ reduced from 0.113 to 0.074 kN/mm. The decrease in *K*_*h*_ with increasing shear strain level can be attributed to decreases in contact area. Including a single steel mesh layer has reduced the *K*_*h*_ of the isolator by 34.5% (at 100% shear strain) and increased the *K*_*v*_ by almost 16%. As the primary shape factor increased from 0.58 to 1.16, the lateral stiffness decreased from 0.155 to 0.113 kN/mm (Fig. 10c). Meanwhile, the damping ratio increased from 8.37 to 11.10% (Fig. 10d). Figure 7 shows the pictorial view of the isolators at 50% and 100% shear strains. For the 50% shear strain, the isolator has not shown any form of internal deformation. At 100% shear strain, debonding was observed at the edge of the isolator, where it is touching the upper surface, and a slight sliding was observed at the edge, where it was touching the bottom surface. Due to the shear strain, the steel woven mesh wire strings get detached at the edges; the sliding occurs in the isolator (see Fig. 8). Even with this minor failure, the behavior of the isolator seems to be effective as the stiffness ratio (*K*_*v*_*/K*_*h*_) obtained for this 2-layer UMELI is 319.

### 3-layer UMELI

**Fig. 9:**
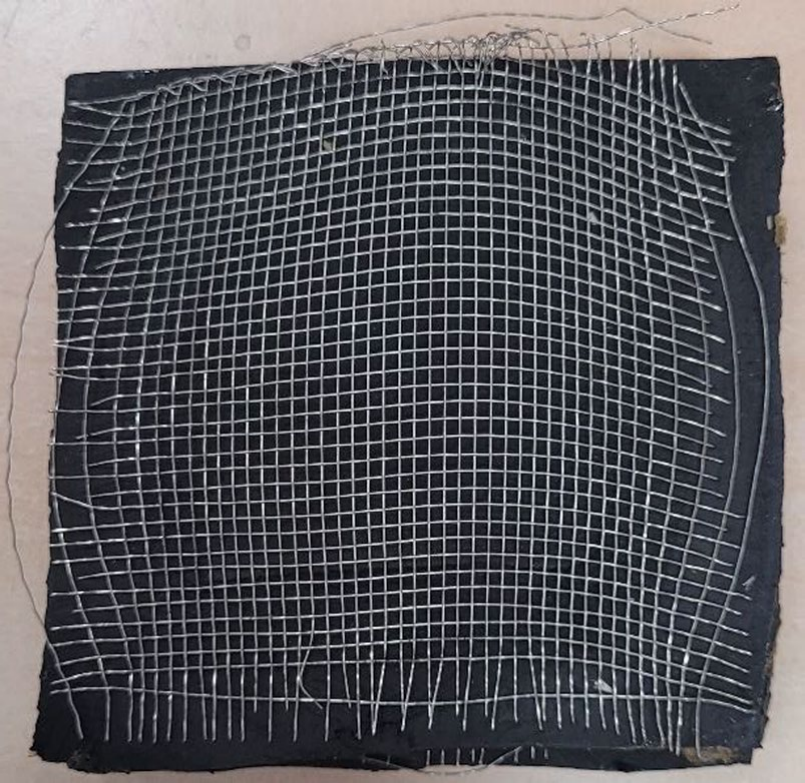
Debonding of the steel mesh wire.

**Fig. 10:**
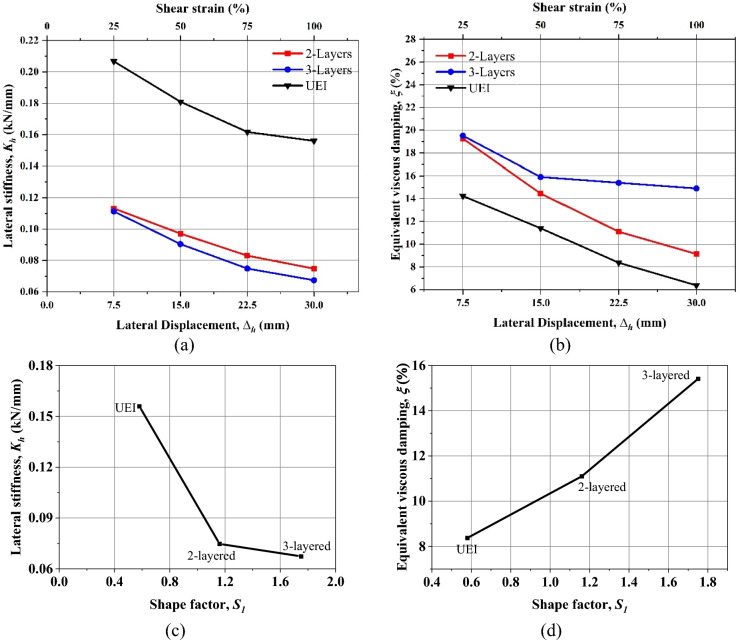
(**a**) Variation of lateral stiffness with respect to shear strain, (**b**) Variation of equivalent viscous damping with respect to shear strain; (**c**) Variation of lateral stiffness with shape factor at 100% shear strain. (**d**) Variation of equivalent viscous damping with shape factor at 100% shear strain.

The shear cyclic test of 3-layer UMELI reveals a decrease in *K*_*h*_ as the shear strain increases. A similar trend was observed in the *ξ* (Fig. 10a,b). The *ξ* decreased from 19.51 to 14.90%, while the *K*_*h*_ decreased from 0.111 to 0.067 kN/mm. The addition of a two-steel mesh layer has resulted in a 56.8% decrease in the *K*_*h*_ of the isolator (at 100% shear strain) and a nearly 47.2% increase in vertical stiffness compared to UEI. As the primary shape factor increased from 0.58 to 1.75, the lateral stiffness decreased from 0.155 to 0.067 kN/mm (Fig. 10c). Concurrently, the damping ratio increased from 8.37 to 15.50% (Fig. 10d). Figure 8 depicts the visual representation of the isolators at 50% and 100% shear strains. The isolator has not exhibited any internal deformation for the 50% shear strain. Debonding occurred at the edge of the isolator when it experienced a shear strain of 100%. This debonding was observed between the second and third layers. Additionally, sliding was observed between the first layer and the second layer. Similar to the 2-layer UMELI, the steel woven mesh wire strings become separated at the edges, causing sliding to occur in the isolator (refer to Fig. 9). Despite this setback, the performance of the isolator appears to be efficient, as evidenced by the stiffness ratio (*K*_*v*_*/K*_*h*_) of 562 achieved for this 3-layer UMELI.

**Fig. 11:**
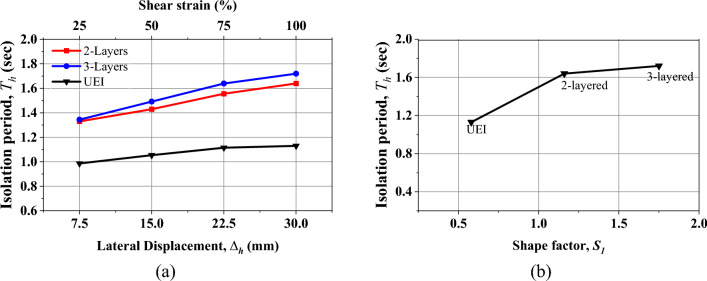
(**a**) Variation of the isolation time period of the isolators with respect to shear strain; (**b**) Variation of the isolation time period of the isolators with respect to shape factor.

Considering a vertical design load of 50 kN, the variation of the fundamental time period is shown in Fig. 11a. This time period is obtained based on the values of effective horizontal stiffness obtained from the experiment. The effective area of the isolator is considered when calculating the isolation period. As seen in Fig. 11a, after 50% shear strain, the time period remains almost constant for all the cases. Since the lateral stiffness of the UMELI decreases with the addition of the steel mesh, the isolation time period also increases. The isolation time period of UEI for 100% shear strain is obtained as 1.13 s. Nonetheless, 1.6 s under 50 kN of vertical load was the initial design period that was considered for 25% strain. The design equations used could not accurately replicate the behavior of the proposed technique, as they predicted the same behavior as U-FREI. Since this technique does not involve bonding between the elastomer and steel mesh and internal sliding within the steel mesh is observed, the current expressions require modification. Addressing these discrepancies is left for future research in this study. Meanwhile, for the UMELI—2 layers, the isolation time period reached 1.72 s. Similarly, for UMELI—3 layers, the isolation period is found to be 1.64 s. The variation of the isolation period with respect to the shape factor is shown in Fig. 11b. Hence, the addition of the steel mesh between the rubber layers has improved the isolation time period (Figs. 10, 11).

From the experimental investigations, the performance of UMELI is superior to that of the UEI. The UMELI offers higher dynamic vertical stiffness than UEI. A significantly larger isolation period and higher energy dissipation capacity were achieved compared to the UEI. Even though sliding of the layer were observed due to the detachment of the steel mesh wire, this detachment of the wires can be prevented by using the welded mesh. Layer separation has been observed at 100% strain and can be anticipated at higher strain levels as well. However, as previously mentioned, the isolator returns to its original position after each cycle without causing permanent dislocation of the layers. At lower strain levels, the layers remain intact despite numerous cyclic reversals, without any permanent dislocation within the isolator. Still, at higher strain levels, permanent dislocation may occur. To prevent this, the isolator displacement should be limited to a specific strain level. Therefore, separation of layers is permissible up to the point of permanent dislocation; beyond this, the technique may be considered unsuitable. Nevertheless, until 100% shear strain, UMELI has proven effective and has higher efficacy than UEI. Still, the performance of UMELI under higher shear strains and with an increased number of steel mesh layers need to carried out, alongside conducting a stability analysis. A design expression needs to be developed that considers frictional interaction and internal sliding, enabling the proposal of upper limits for the technique to avoid the permanent deformation. Additionally, shake table testing of the masonry model with UMELI needs to be performed.

## Conclusion

The primary objective of this study is to evaluate the effectiveness of an unbonded mesh elastomeric layered isolator (UMELI) as a low-cost isolation technique for masonry structures. The isolators are investigated for their effectiveness in terms of equivalent lateral stiffness, equivalent viscous damping ratio, and vertical stiffness. Using the initial design, the isolators were procured and assembled. It was then tested for its mechanical properties. The response characteristics of the UMELI with varying steel mesh layers were evaluated and compared with those of an unreinforced elastomeric isolator (UEI). All of the isolator's thicknesses were normalized as the addition of the steel mesh layer increased the thickness of UMELI. The key findings of the study is summarized below.

1. The vertical stiffness of the 2-layered UMELI is found to be around 16% higher than the UEI. Whereas 3-layered UMELI has 47% higher stiffness than the UEI.
2. The vertical frequency of 2-layered and 3-layered UMELI is 8% and 27% higher than the UEI. This frequency is higher than the frequencies, which causes rocking instability.
3. At 100% shear strain, UMELI has 52–56% greater lateral flexibility compared to the UEI.
4. At 100% shear strain, the isolation period of UMELI is 30–34% longer than the UEI.
5. The effective damping ratio of UMELI is found to be larger than the UEI.

The proposed UMELI is shown effectiveness till 100% shear strain with two and three layer elastomers. Further, the performance of UMELI with higher shear strains and an increased number of steel mesh layers needs to be assessed. In addition to that, shake table testing of the masonry model with the UMELI needs to be studied in detail.

## Data Availability

The datasets used and/or analyzed during the current investigation are available from the corresponding author upon reasonable request.

## References

[1] Rota, M., Penna, A. & Magenes, G. A methodology for deriving analytical fragility curves for masonry buildings based on stochastic nonlinear analyses. *Eng. Struct.* **32**(5), 1312–1323 (2010). 10.1016/j.engstruct.2010.01.009

[2] Sar, D., & Sarkar, P. (2013). Seismic evaluation of existing unreinforced masonry building. in *Proceedings of the International Symposium on Engineering under Uncertainty: Safety Assessment and Management (ISEUSAM-2012)* (pp. 1267–1276). Springer India.

[3] Sarkar, A., Halder, L., & Sharma, R. P. (2015). Seismic damage evaluation of unreinforced masonry buildings in high seismic zone using the nonlinear static method. in *Advances in Structural Engineering: Dynamics*, Vol. 2 (pp. 1039–1053). Springer India.

[4] Parisi, F., Augenti, N. & Prota, A. Implications of the spandrel type on the lateral behavior of unreinforced masonry walls. *Earthquake Eng. Struct. Dynam.* **43**(12), 1867–1887 (2014). 10.1002/eqe.2441

[5] Erberik, M. A. Generation of fragility curves for Turkish masonry buildings considering in-plane failure modes. *Earthquake Eng. Struct. Dynam.* **37**(3), 387–405 (2008). 10.1002/eqe.760

[6] Bothara, J. K., Dhakal, R. P. & Mander, J. B. Seismic performance of an unreinforced masonry building: An experimental investigation. *Earthquake Eng. Struct. Dynam.* **39**(1), 45–68 (2010). 10.1002/eqe.932

[7] Parisi, F. & Augenti, N. Assessment of unreinforced masonry cross sections under eccentric compression accounting for strain softening. *Constr. Build. Mater.* **41**, 654–664 (2013). 10.1016/j.conbuildmat.2012.12.039

[8] Prasad, J. S. R., Singh, Y., Kaynia, A. M. & Lindholm, C. Socioeconomic clustering in seismic risk assessment of urban housing stock. *Earthq. Spectra* **25**(3), 619–641 (2009). 10.1193/1.3158547

[9] Di Sarno, L. & Elnashai, A. *Fundamentals of Earthquake Engineering* (Wiley, 2008).

[10] Garevski, M. Development, production, and implementation of low-cost rubber bearings. In *Geological and Earthquake Engineering*, Vol. 17, 411–437 (Springer, 2010)

[11] Braga, F., Faggella, M., Gigliotti, R. & Laterza, M. Nonlinear dynamic response of HDRB and hybrid HDRB-friction sliders base isolation systems. *Bull. Earthq. Eng.* **3**, 333–353 (2005). 10.1007/s10518-005-1242-2

[12] Clemente, P. *et al.* Effectiveness of HDRB isolation systems under low energy earthquakes. *Soil Dyn. Earthq. Eng.* **118**, 207–220 (2019). 10.1016/j.soildyn.2018.12.018

[13] Di Sarno, L., Chioccarelli, E. & Cosenza, E. Seismic response analysis of an irregular base isolated building. *Bull. Earthq. Eng.* **9**, 1673–1702 (2011). 10.1007/s10518-011-9267-1

[14] Dolce, M., Cardone, D., Ponzo, F. C. & Valente, C. Shaking table tests on reinforced concrete frames without and with passive control systems. *Earthquake Eng. Struct. Dynam.* **34**(14), 1687–1717 (2005). 10.1002/eqe.501

[15] Makris, N. Seismic isolation: Early history. *Earthquake Eng. Struct. Dynam.* **48**(2), 269–283 (2019). 10.1002/eqe.3124

[16] Mayes, R. L., & Naeim, F. (2001). Design of structures with seismic isolation. in *The Seismic Design Handbook*, 723–755.

[17] Mazza, F. & Vulcano, A. Nonlinear response of RC framed buildings with isolation and supplemental damping at the base subjected to near-fault earthquakes. *J. Earthquake Eng.* **13**(5), 690–715 (2009). 10.1080/13632460802632302

[18] Toopchi-Nezhad, H., Tait, M. J. & Drysdale, R. G. Testing and modeling of square carbon fiber-reinforced elastomeric seismic isolators. *Struct. Control Health Monit. Off. J. Int. Assoc. Struct. Control Monit. Eur. Assoc. Control Struct.* **15**(6), 876–900 (2008).

[19] Toopchi-Nezhad, H., Tait, M. J. & Drysdale, R. G. Lateral response evaluation of fiber-reinforced neoprene seismic isolators utilized in an unbonded application. *J. Struct. Eng.* **134**(10), 1627–1637 (2008). 10.1061/(ASCE)0733-9445(2008)134:10(1627)

[20] Spizzuoco, M., Calabrese, A. & Serino, G. Innovative low-cost recycled rubber–fiber reinforced isolator: Experimental tests and finite element analyses. *Eng. Struct.* **76**, 99–111 (2014). 10.1016/j.engstruct.2014.07.001

[21] Sierra, I. E. M., Losanno, D., Strano, S., Marulanda, J. & Thomson, P. Development and experimental behavior of HDR seismic isolators for low-rise residential buildings. *Eng. Struct.* **183**, 894–906 (2019). 10.1016/j.engstruct.2019.01.037

[22] Ghorbi, E. & Toopchi-Nezhad, H. Annular fiber-reinforced elastomeric bearings for seismic isolation of lightweight structures. *Soil Dynam. Earthq. Eng.* **166**, 107764 (2023). 10.1016/j.soildyn.2023.107764

[23] Ghorbi, E., & Toopchinezhad, H. Seismic isolation of a residential masonry building with annular unbonded elastomeric isolators.

[24] Calabrese, A., Losanno, D., Spizzuoco, M., Strano, S. & Terzo, M. Recycled Rubber Fiber Reinforced Bearings (RR-FRBs) as base isolators for residential buildings in developing countries: The demonstration building of Pasir Badak, Indonesia. *Eng. Struct.* **192**, 126–144 (2019). 10.1016/j.engstruct.2019.04.076

[25] Turer, A. & Özden, B. Seismic base isolation using low-cost Scrap Tire Pads (STP). *Mater. Struct.* **41**, 891–908 (2008). 10.1617/s11527-007-9292-3

[26] Masoudi, M., & Ghalehnoy, M. (2018). Seismic response of base-isolated structures with insufficient gaps. in *16th European conference on earthquake engineering, Thessaloniki, Greece*.

[27] Md, Z. N., Mohan, S. C. & Jyosyula, S. K. R. Development of low-cost base isolation technique using multi-criteria optimization and its application to masonry building. *Soil Dynam. Earthq. Eng.* **172**, 108024 (2023). 10.1016/j.soildyn.2023.108024

[28] Md, Z. N., Mohan, S. C., & Jyosyula, S. K. R. (2024). Performance of low-cost unreinforced elastomeric isolator for masonry building: Experimental investigations and numerical analysis. in *Structures* (Vol. 63, p. 106365). Elsevier.

[29] Zoheb, N. M., Mohan, S. C., & Kalyana Rama, J. S. (2022). Seismic behavior of masonry building with spread base isolation using natural rubber. in *Lifelines* 2022 (pp. 556–565).

[30] Li, H., Tian, S., Dang, X., Yuan, W. & Wei, K. Performance of steel mesh reinforced elastomeric isolation bearing: Experimental study. *Construct. Build. Mater.* **121**, 60–68 (2016). 10.1016/j.conbuildmat.2016.05.143

[31] Karimi, A. A., Toopchi-Nezhad, H. & Memarzadeh, P. Performance of novel rectangular partially bonded steel mesh-reinforced elastomeric bearings for seismic isolation of bridges. *J. Bridge Eng.* **29**(7), 04024042 (2024). 10.1061/JBENF2.BEENG-6563

[32] Losanno, D., Ravichandran, N. & Parisi, F. Seismic fragility models for base-isolated unreinforced masonry buildings with fibre-reinforced elastomeric isolators. *Earthquake Eng. Struct. Dynam.* **52**(2), 308–334 (2023). 10.1002/eqe.3761

[33] Kelly, J. M. *Earthquake-Resistant Design with Rubber* Vol. 7 (Springer-Verlag, 1993).

[34] Tsai, H. C. & Kelly, J. M. Buckling of short beams with warping effect included. *Int. J. Solids Struct.* **42**(1), 239–253 (2005). 10.1016/j.ijsolstr.2004.07.021

[35] Russo, G., Pauletta, M. & Cortesia, A. A study on experimental shear behavior of fiber-reinforced elastomeric isolators with various fiber layouts, elastomers and aging conditions. *Eng. Struct.* **52**, 422–433 (2013). 10.1016/j.engstruct.2013.02.034

[36] Toopchi-Nezhad, H. Horizontal stiffness solutions for unbonded fiber reinforced elastomeric bearings. *Struct. Eng. Mech. Int. J.* **49**(3), 395–410 (2014). 10.12989/sem.2014.49.3.395

[37] Kelly, J. M. Analysis of fiber-reinforced elastomeric isolators. *J. Seismol. Earthquake Eng.* **2**(1), 19–34 (1999).

[38] De Raaf, M. G., Tait, M. J. & Toopchi-Nezhad, H. Stability of fiber-reinforced elastomeric bearings in an unbonded application. *J. Compos. Mater.* **45**(18), 1873–1884 (2011). 10.1177/0021998310388319

[39] Galano, S., Losanno, D. & Calabrese, A. Stability analysis of unbonded fiber reinforced isolators of square shape. *Eng. Struct.* **245**, 112846 (2021). 10.1016/j.engstruct.2021.112846

[40] Tran, C., Calabrese, A., Vassiliou, M. F. & Galano, S. A simple strategy to tune the lateral response of unbonded Fiber Reinforced Elastomeric Isolators (FREIs). *Eng. Struct.* **222**, 111128 (2020). 10.1016/j.engstruct.2020.111128

[41] Osgooei, P. M., Van Engelen, N. C., Konstantinidis, D. & Tait, M. J. Experimental and finite element study on the lateral response of modified rectangular fiber-reinforced elastomeric isolators (MR-FREIs). *Eng. Struct.* **85**, 293–303 (2015). 10.1016/j.engstruct.2014.11.037

